# Distinguishing the Unique Neuropathological Profile of Blast Polytrauma

**DOI:** 10.1155/2017/5175249

**Published:** 2017-03-23

**Authors:** W. Brad Hubbard, Shaylen Greenberg, Carly Norris, Joseph Eck, Erin Lavik, Pamela VandeVord

**Affiliations:** ^1^Biomedical Engineering and Mechanics, Virginia Tech, Blacksburg, VA 24061, USA; ^2^Translational Biology, Medicine, and Health, Virginia Tech, Blacksburg, VA 24061, USA; ^3^Department of Chemical, Biochemical and Environmental Engineering, University of Maryland, Baltimore County, Baltimore, MD 21250, USA; ^4^Research Services, Salem VAMC, Salem, VA 24153, USA

## Abstract

Traumatic brain injury sustained after blast exposure (blast-induced TBI) has recently been documented as a growing issue for military personnel. Incidence of injury to organs such as the lungs has decreased, though current epidemiology still causes a great public health burden. In addition, unprotected civilians sustain primary blast lung injury (PBLI) at alarming rates. Often, mild-to-moderate cases of PBLI are survivable with medical intervention, which creates a growing population of survivors of blast-induced polytrauma (BPT) with symptoms from blast-induced mild TBI (mTBI). Currently, there is a lack of preclinical models simulating BPT, which is crucial to identifying unique injury mechanisms of BPT and its management. To meet this need, our group characterized a rodent model of BPT and compared results to a blast-induced mTBI model. Open field (OF) performance trials were performed on rodents at 7 days after injury. Immunohistochemistry was performed to evaluate cellular outcome at day seven following BPT. Levels of reactive astrocytes (GFAP), apoptosis (cleaved caspase-3 expression), and vascular damage (SMI-71) were significantly elevated in BPT compared to blast-induced mTBI. Downstream markers of hypoxia (HIF-1*α* and VEGF) were higher only after BPT. This study highlights the need for unique therapeutics and prehospital management when handling BPT.

## 1. Introduction

A traumatic event that causes multiple injuries, or polytrauma, is a complex challenge for clinicians [1]. Recently, polytrauma has been reported in military populations with a direct link to blast exposure [2]. A rise in terrorism worldwide also fuels the polytrauma epidemic for civilian casualties. Reports from terrorist activity in the Middle East and Europe in the late twentieth century highlight the growing issues and prevalence of primary blast lung injury (PBLI) [3–9]. With the increased use of improvised explosive devices (IEDs) in warfare, blast loading produces debilitating effects on victims from military conflicts and acts of terrorism [10, 11].

Blast-induced polytrauma (BPT) poses a unique obstacle to physicians due to the complex systematic interactions. Diagnosing traumatic brain injuries (TBIs) is a significant task as the amount of concomitant injuries overshadows signs of neurotrauma. There is limited knowledge regarding the early stage management of polytrauma and the sensitive underlying systemic mechanisms that contribute to ongoing neuropathology. Injuries, such as pneumothorax or uncontrolled bleeding, take precedence in early stage trauma care. While these concerns are severe and need immediate treatment, TBIs often cause the most long-term harm to the surviving victims of blast exposure. In order to guide trauma management, as well as initial treatment for TBI, a preclinical model characterizing the intricate aspects of polytrauma is needed. McDonald et al. reported evidence that additional injuries, concomitant to impact-related TBI, can increase both peripheral and central inflammatory response as well as exacerbate TBI pathology [12]. We expect that BPT models will show exacerbated vascular and inflammatory neuropathology due to these concomitant injuries.

A major objective of this study was to assess the role that subsequent hypoxia after blast exposure has on the outcomes of blast-induced TBI. Downstream neurological regulators of hypoxia and blood-brain barrier (BBB) disruption were examined as critical measures. Hypoxia likely contributes to exacerbating the injury progression after BPT due to impairment of pulmonary gas exchange in the lung, resulting in secondary effects on cerebral vasculature [13, 14]. Primary blast exposure to the brain also causes BBB disruption leading to a myriad of molecular cascades [15]. This cyclical relationship is highlighted with the finding that BBB disruption following TBI is biphasic, occurring at multiple time points after injury [16]. Comparing our BPT model to an established blast-induced mTBI model allows for the elucidation of molecular pathways triggered by the additive hypoxic environment. Examining how systemic pathology after lung injury impacts neuropathology is crucial to understanding mechanisms of blast-induced polytrauma and these results will aid in the development of injury-specific pharmacological targets that may be more effective in treating BPT.

## 2. Materials and Methods

### 2.1. Experimental Set-Up

All the experiments are in accordance with the Virginia Tech Institutional Animal Care and Use Committee and all the experimental protocols described herein have been approved. Prior to all experiments, male Sprague Dawley rats (~325 g, Harlan Labs, San Diego) were acclimated to a 12-hour light/dark cycle with food and water provided ad lib. As described previously, the shock front static and dynamic overpressures were generated using a custom-built Advanced Blast Simulator (200 cm × 30.48 cm × 30.48 cm) that consists of a driving compression chamber attached to a rectangular transition and testing chamber with an end wave eliminator (EWE) (ORA Inc., Fredericksburg, VA) located at the Center for Injury Biomechanics of Virginia Tech University. The passive EWE, installed at the venting end of the ABS, minimizes the shock wave outflow by means of a specially designed plate system. Patterns in the EWE plate system were created to mirror reflected shocks and rarefactions, which tend to cancel each other out and diminish unwanted effects within the test section. A peak static overpressure was produced with compressed helium and calibrated acetate sheets (Grafix Plastics, Cleveland, OH). Three pressure measurements were collected at 250 kHz using a Dash 8HF data acquisition system (Astro-Med, Inc., West Warwick, RI) and peak overpressures were calculated by determining wave speed (m/s) at the specimen position. A mesh sling, used to hold the animal during the exposure, allowed for minimal hindrance of the wave through the chamber. Shock wave profiles were verified to maintain consistent exposure pressures between subjects. Animals from the mTBI group were anesthetized with 3% isoflurane before being placed in a rostral cephalic orientation towards the shock wave. Whole body exposure is considered “on-axis” with the animal facing rostral cephalic orientation towards the blast. This exposure has minimal effect on the lungs, as the shock streamlines around the body. Thus, resulting exposure in this study creates a relatively specific brain injury and minimal polyorgan trauma. The mTBI rodent group was exposed to a single incident pressure profile resembling a “free-field” blast exposure, single Friedlander-like waveform that is in mild-moderate range at 117 kPa (17 psi) with a positive duration of 2.5 ms.

For the BPT group, rats were anesthetized with a ketamine/xylazine solution, in accordance with the rodent weight, for sedation during blast. BPT animals were exposed to a single incident pressure profile resembling a “free-field” blast exposure at a range of 170 to 210 kPa (24.5 to 30.5 psi) peak overpressure with 2.5 ms positive phase duration to ensure severe levels of PBLI [17, 18]. Rodents in the BPT group were positioned in a prone orientation with the right side of the thorax facing the shock front. The animals were not allowed to impact any solid surface in order to prevent secondary injuries and this was confirmed using high-speed video (Phantom Miro eX2, Vision Research). All animals were randomly assigned to one of three groups: mTBI, BPT, and sham (*n* = 8–12/group). Sham animals underwent all procedures, including ketamine/xylazine sedation, as the BPT group except for blast exposure.

### 2.2. Open Field Test

Seven days after injury, animals performed an open field thigmotaxis assessment [19, 20]. Briefly, an opaque black acrylic box with dimensions 80 × 80 × 36 cm was used for the task. Animals were acclimated in the open field box before the injury and two days after injury. The acclimation ensures that any anxiety-like traits would be due to the blast and subsequent injury progression. Activity changes were detected using EthoVision XT™ software tracking. Thigmotaxia, or the animal's preference of proximity to the arena walls, tends to decrease after a period of acclimation but is continuously displayed in animals with anxiety. Time spent along the chamber wall reflects an increased level of anxiety and is a common method of determining anxiety levels [20]. Rats were videotaped for five minutes and avoidance of center square activity (i.e., anxiety-related behavior) was measured by determining the amount of time and frequency of entries into the central portion of the open field.

### 2.3. Tissue Processing

After seven days, animals were euthanized by transcardial perfusion of saline and 4% paraformaldehyde. Following collection, brains were stored in a 4% paraformaldehyde fixative solution. After 48 hours in fixative, whole brains were placed in 30% sucrose solution for tissue sectioning preparation. Whole brains were embedded in Tissue-Tek® optimal cutting temperature embedding medium (Sakura Finetek USA, Inc., Torrance, CA) for cryostat processing in the coronal plane. Samples were then cut (40 *μ*m) and sections containing amygdala nuclei were isolated (bregma: −2.28 mm).

### 2.4. Immunofluorescent Staining

Immunohistochemistry was performed on amygdalar sections to evaluate levels of markers: glial fibrillary acidic protein (GFAP), cleaved caspase-3, ionized calcium-binding adaptor molecule 1 (IBA-1), SMI-71, hypoxia inducible factor-1*α* (HIF-1*α*), and vascular endothelial growth factor (VEGF). Samples were rinsed three times with PBS and incubated in 2% bovine serum albumin (BSA) in PBS for one hour at room temperature. Sections were then incubated with a primary antibody: anti-GFAP (1 : 500; Invitrogen, Carlsbad, California), anti-caspase-3 (1 : 500; Cell Signaling Technologies, Danvers, Massachusetts), anti-IBA-1 (1 : 500; Biocare Medical, Concord, California), anti-SMI-71 (1 : 250; Covance, Princeton, New Jersey), anti-HIF-1*α* (1 : 250; Novus Biologicals, Littleton, Colorado), or anti-VEGF (1 : 250; Santa Cruz, Dallas, Texas) overnight at 4°C. Primary antibodies were labeled separately on different amygdalar sections. After a PBS wash, the samples were incubated for 1.5 hours with fluorescein isothiocyanate (FITC) anti-rat, Alexa Fluor 555 anti-rabbit, Alexa Fluor 488 anti-mouse, or Alexa Fluor 594 anti-mouse. After three PBS washes (five minutes each), samples were mounted, air-dried, and coverslipped with ProLong Antifade Gold Reagent with 4′,6-diamidino-2-phenylindole (DAPI; Invitrogen, Carlsbad, CA). Sections were examined under a Zeiss fluorescence microscope at 20x magnification under appropriate fluorescent filters and images were taken by Zeiss AxioCam ICc 1. For all images, quantification (ImageJ software; NIH, Bethesda, MD) was based on fluorescence intensity after thresholding to eliminate background color. For average intensity, the output variable corresponds to the average fluorescent intensity per pixel (a number between 0 and 255). Percent area gives an indication of the amounts of pixels with signal divided by the total amount of pixels.

### 2.5. Statistical Analysis

Statistical differences between the treatment groups were assessed with analysis of variance, or ANOVA, using LSD post hoc test. All statistical analysis was performed using JMP Pro 10 (SAS Institute, Cary, NC) and *p* < 0.05 was considered statistically significant. Unless indicated otherwise, data are presented as mean ± standard error of the mean, or SEM.

## 3. Results

### 3.1. Anxiety Assessment

**Figure 1:**
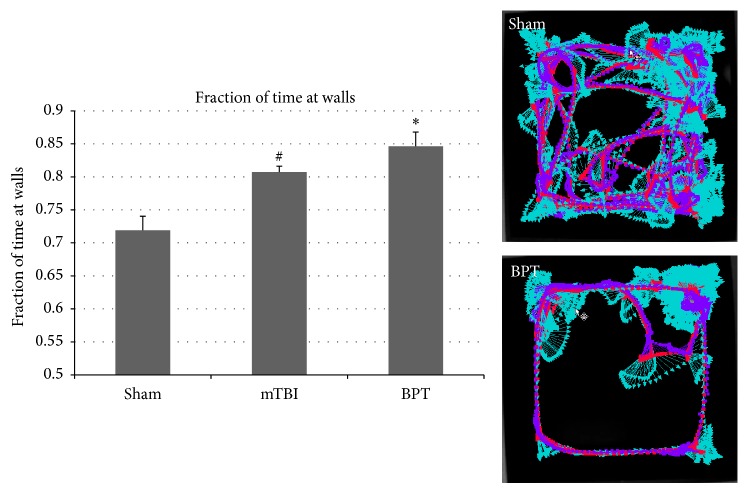
Fraction of time spent at the walls of the open arena was significantly higher in the mTBI and BPT groups compared to sham at 7 days after blast (^*∗*^*p* < 0.02, ^#^*p* < 0.05). Representative images show animal tracking over five minutes.

The fraction of time spent at the walls of the open field box for the BPT group was significantly increased (*p* < 0.02) compared to sham (Figure 1). The mTBI group also displayed elevated anxiety (*p* < 0.05) compared to sham. Representative image of animal activity over the five-minute period in the open arena demonstrates global exploration by the sham group and proximity to the walls in the BPT group (Figure 1). This display of anxiety-like behavior in the BPT group could be the neurological manifestation of injury pathology.

### 3.2. Astrocyte Activation

**Figure 2:**
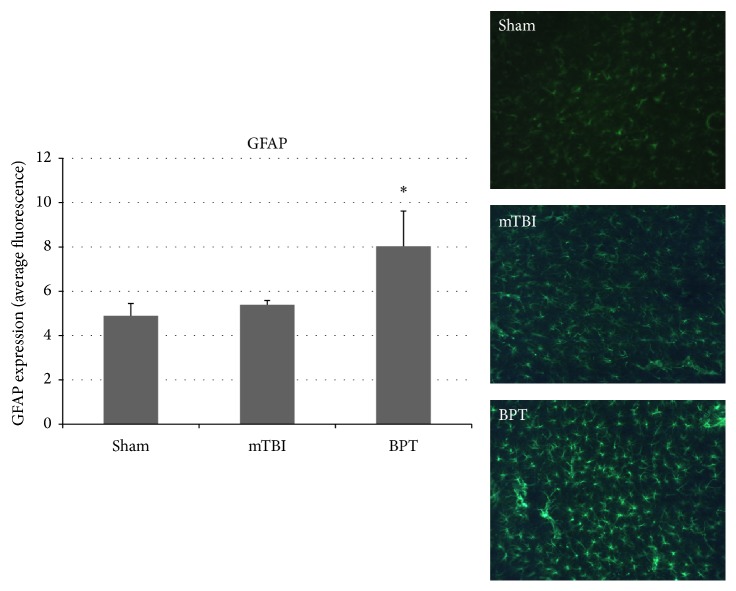
Representative images show reactive glia present seven days after blast in the BPT group. GFAP expression, examining astrocytosis, was significantly elevated in the BPT group compared to sham (^*∗*^*p* < 0.05).

GFAP is a reliable marker to examine astrocyte morphology, as it is constitutively expressed in astrocytes. Higher expression of GFAP is seen in reactive astrocytes and is a standard method to assess astrogliosis [21]. While only slight elevation is seen in the mTBI group compared to sham, the BPT group is significantly different compared to sham (Figure 2). Images show astrocyte populations in each group, though more GFAP expression due to astrogliosis is seen qualitatively in the BPT group.

### 3.3. Apoptosis

**Figure 3:**
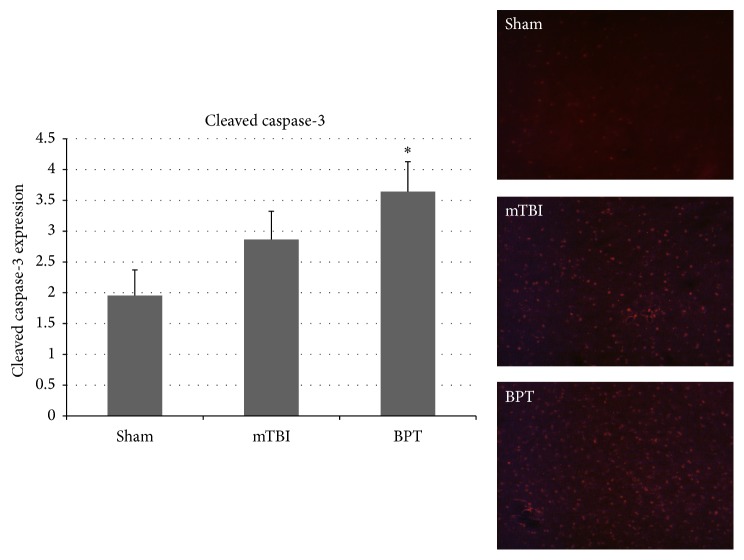
Representative images depict higher number of cells undergoing apoptosis in the mTBI and BPT groups. Cleaved caspase-3 expression was significantly elevated in the BPT compared to the sham group (^*∗*^*p* < 0.05). mTBI group are elevated compared to sham, though not significant (*p* < 0.24).

Cleaved caspase-3 is a protein that is expressed in cells undergoing apoptotic signaling events, which makes it a reliable marker for apoptosis. Expression of cleaved caspase-3 was elevated in both blast groups, although only significantly different in the BPT group (Figure 3). Amygdalar images show an elevated number of apoptotic cells in the blast groups compared to sham.

### 3.4. Microglia Activation

**Figure 4:**
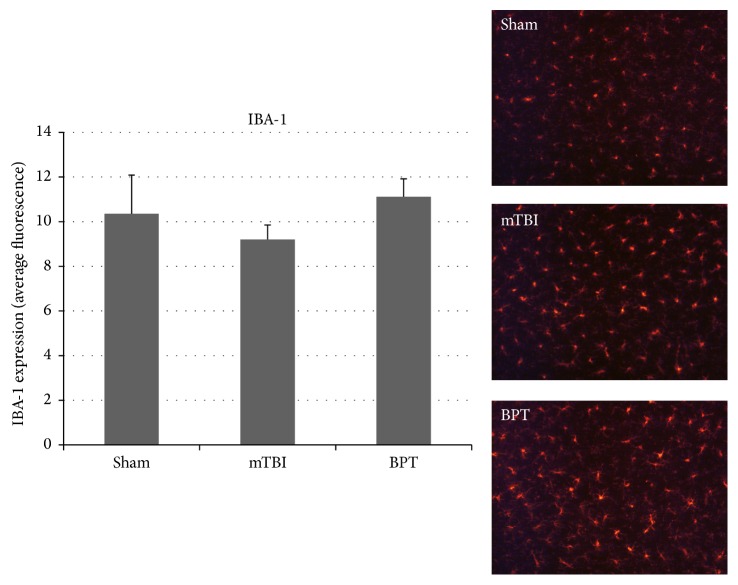
Images show similar number of microglia. IBA-1 expression, marking microglia, in the amygdala was not significantly different between groups.

IBA-1, which is involved in phagocytosis and actin reorganization in microglia, is constitutively expressed in microglia. Though not specific to activated microglia, IBA-1 is usually used to assess morphology (ramified or ameboid), which gives information about microglial modulation in disease states. Although there is no significant difference between the BPT group and sham, there is slight elevation in IBA-1 expression in the BPT group compared to sham (Figure 4).

### 3.5. Blood-Brain Barrier Disruption

**Figure 5:**
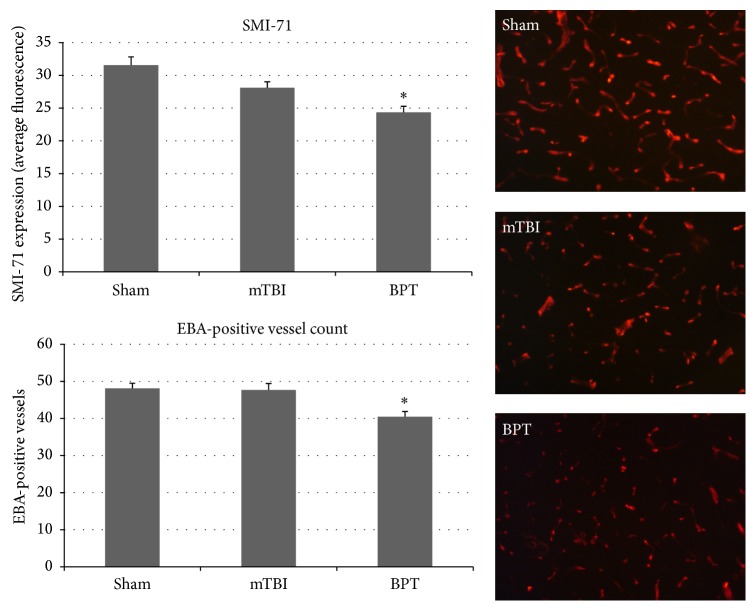
Representative images show lower number of vessels with EBA (BBB competent) in the BPT group. SMI-71 average fluorescence and marking of EBA+ vessels both show significant decrease in the BPT group compared to sham (^*∗*^*p* < 0.001).

SMI-71 is an established antibody against rat endothelial barrier antigen [22, 23]. This antibody binds to EBA, which is not present in vessels with BBB disruption [23].  Figure 5 depicts the decreased staining found within the injury group compared to sham due to decrease in vessel count with EBA expressed. The expression of SMI-71 was decreased in the BPT group, which has been shown previously to signify a compromised BBB [24], compared to the sham group (*p* value < 0.001).

### 3.6. HIF-1*α*

**Figure 6:**
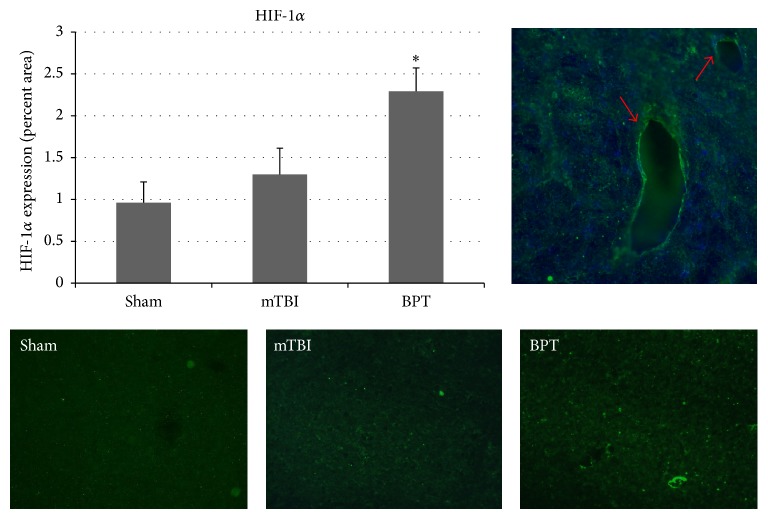
Representative images show HIF-1*α* expression is elevated in the BPT group (^*∗*^*p* < 0.05) when compared to the sham group. (Top right) HIF-1*α* was colocalized with DAPI around the vessels in the BPT group.

HIF-1*α* is a transcription factor that is involved in several injury modalities where hypoxia occurs, including TBI [25]. HIFs are heterodimeric transcription factors composed of an oxygen-sensitive *α*-subunit and a constitutively expressed *β*-subunit. Under normoxia, the HIF-1*α* subunit is constitutively transcribed but constantly targeted for degradation. As oxygen tension drops, the degradation enzymes are inhibited, which results in cytoplasmic stabilization of the *α*-subunits. For the BPT group, HIF-1*α* expression was increased in the amygdala at seven days after blast compared to the sham group (Figure 6). In Figure 6, HIF-1*α* appears to be colocalized with DAPI around major vessels, showing that hypoxia is potentially being sensed first due to low blood oxygen concentration and this could be an ongoing mechanism.

### 3.7. VEGF Expression

**Figure 7:**
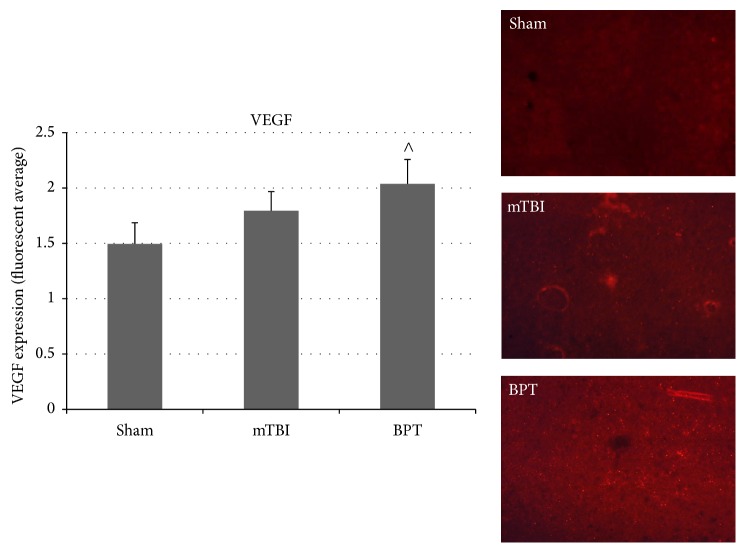
VEGF expression in the amygdala of the BPT group was elevated over sham, though not significant (^∧^*p* = 0.064). Representative images depict VEGF expression in the amygdala.

VEGF, a signal protein, is produced to exert angiogenic stimulation. VEGF usually has a downstream role in response to HIF-1*α* transcription in hypoxic cells [26]. Overexpression of VEGF has been shown to contribute to neurological disease [27–29]. Expression of VEGF was elevated though not statistically different in the BPT group compared to sham (Figure 7).

## 4. Discussion

### 4.1. Polytrauma Rodent Model

Preclinical models designed for investigation of lung injury and neurotrauma sustained from blast exposure are scarce in the literature [18, 30]. As such, there is a lack of identifiable polytraumatic-specific injury markers for clinical use. Primary blast exposure has been correlated with varying TBI injury severities with assessment of physiology and lung injury in the rodent model shown by Mishra et al. [31]. BBB damage, signified by immunoglobulin G (IgG), has been characterized following blast trauma but exact mechanisms and time of BBB repair have not been elucidated [30]. In a lateral/side-on blast exposure to unanesthetized rodents, pulmonary hemorrhage was reported after 116 kPa exposure in addition to motor function impairment with an absence of axonal injury [32]. Another BPT model was created by exposing the animal to a blast wave directed at the thorax [33]. This model relied on the “vascular pulse” blast injury mechanism where blast overpressure causes pressure differentials in vasculature and produces a wave to the cerebrovasculature. Lung injury and perivenular neuroinflammation were found in this study [33], highlighting the importance of systemic circulation in polytrauma. Our model has been previously characterized and blast-induced lung injury mechanisms, as well as apparent pathology, have been deduced [17, 18]. Decreased oxygen saturation due to lung injury has been reported acutely, which is the major premise of hypoxia in the current BPT model.

### 4.2. Polytrauma Worsens Behavioral Deficits

The open field test is a standard test to measure anxiety-like behavior [34]. Rodents exposed to BPT displayed more time against the walls of the open field arena, or elevated anxiety-like behavior, compared to other groups. Anxiety has been reported seven to nine days after blast exposure [35, 36]. In a blast-induced TBI model, anxiety was seen in open field activity at seven days after following 25–40 psi blast exposure [37]. In a rat model of blast-induced mTBI, minocycline was administered and negated anxiety seen in injured animals at eight days after injury [38]. The BPT group displays worsened behavior outcome compared to mTBI animals, pointing to neural dysregulation due to systemic influence in the BPT group.

### 4.3. Exacerbated Pathology after Polytrauma

The results showed that our BPT model has unique neuropathological features compared to the blast-induced mTBI model. While similar markers are increased in both injury models, injury markers in general are exacerbated in the polytrauma model. Elevated GFAP expression and cleaved caspase-3 have been reported over the course of multiple time points after blast in the amygdala [39, 40]. Higher expression of GFAP and cleaved caspase-3 in the BPT model shows that there are more astrogliosis and apoptosis with higher injury severity. While differences in IBA-1 are not seen in this model, activation-specific antibodies, such as CD68 or CD11b, could be investigated to assay microglia activation after BPT. An antibody, like CD68, would be more sensitive as it has a distinct role in phagocytosis during the activation process. There is a potential that microglia at seven days after blast are in a retracted process activation state [41] and this morphology would be difficult to quantify with IBA-1.

A major finding was that BBB disruption, highlighted by a reduction in EBA+ vessels at seven days after injury, plays a distinct role in BPT injury pathology. Disruption of the BBB is a common finding in models of polytrauma but the exact mechanisms have not been deduced [30, 31, 33]. This could be a crucial upstream event in an ongoing injury cascade, involving hypoxia and subsequent BBB modulation. Along with secondary mechanisms of neuroinflammation and apoptosis, BBB disruption has been examined in blast-induced mTBI studies and has been reported acutely with recovery at 30 days after blast [42–46]. Hypoxia can produce BBB disruption and increased permeability, according to Kaur and Ling [47]. Clinically, compromise of astrocytic endfeet coverage of blood vessels in the brain has been reported in depressive disorders [48]. Exact blast injury thresholds have yet to be determined to produce consistent BBB breakdown but levels in this study indicated BBB compromise, subsequently leading to debilitating neurological consequence [43, 49]. SMI-71, as a marker for BBB disruption, has been correlated with FITC-albumin infiltration [23]. Lower number of EBA+ vessels and stained vessel area were associated with regions of BBB dysfunction in the BPT group.

In addition to BBB damage that occurs from the primary blast wave, hypoxic conditions are present when lung injury has occurred which contributes to BBB disruption [50]. HIF-1*α* is a transcription factor that is involved in several injury modalities where hypoxia occurs, including TBI [51]. Even though HIF-1*α* has been shown to play a role in TBI progression and cerebral ischemia, few studies have examined its role after mTBI and it has not been investigated in relation to BPT [52, 53]. Delayed opening of the BBB, that is, only after HIF-1*α* was already stabilized, suggested barrier stability is mediated via one or more HIF-1*α* effectors [54]. HIF-1*α* is a mediator of disruption of the BBB and has been shown to have detrimental effects on injury pathology in the brain [55]. Inhibition of HIF-1*α* has been reported to reduce BBB damage and improve recovery from cerebral ischemia in rats [28, 55], possibly by reducing levels of VEGF and attenuating the expression of cleaved caspase-3 and p53, which are key molecules in the apoptosis pathway [56]. Inhibition of VEGF has shown to restore integrity of the BBB after an insult, possibly through modulation of matrix metalloproteinases (MMPs) [27]. VEGF, a downstream factor, can cause a leaky BBB [54]. Overall, BBB disruption is based on many factors during and after hypoxia with enhanced production of VEGF and inflammatory cytokines constituting ongoing pathways [47]. This injury pathway in the amygdala can potentially lead to neurologic impairment, such as anxiety [35]. Characterizing the role of secondary markers in BPT pathology would contribute to understanding of injury pathways, such as BBB dysfunction, and lead to novel therapeutic options.

Even though no significant difference was found in VEGF staining in BPT compared to sham, this factor could still play a role in the pathology at different time points. The role of VEGF, a downstream factor in injury cascades, in BBB disruption has been established in models of brain injury [29, 57]. Preclinical studies of mTBI reported increased levels of VEGF five days after injury in the amygdala [39]. As VEGF is a potential downstream marker after HIF-1*α* presence, it is possible that VEGF has significantly elevated expression after the seven-day time point. After multiple blast exposure, VEGF levels in plasma were upregulated at two hours after multiple injuries but not at 22 days after injury [58]. In a repeated mild blast TBI model, long lasting (42 days after injury) elevated levels of HIF-1*α* and VEGF in plasma were reported and due to hypoxia at time of injury [59]. After severe TBI in a rat model, HIF-1*α* level in serum steadily increased from one day to 28 days after injury, showing delayed response and release into the bloodstream [60]. HIF-1*α* has been shown to play a distinct role in apoptosis and BBB disruption after TBI in several models [51, 61]. Acute presentation of hypoxic factors would validate the findings of secondary mechanisms of hypoxic insult to the amygdala seven days after blast. More studies need to be conducted to fill in these knowledge gaps.

### 4.4. Mechanical Damage to the Brain by the Blast Wave

The mechanics of how primary blast exposure specifically injures the brain, usually in an inhomogeneous way, are poorly understood and are likely to be dependent upon orientation to the blast. Since the BPT model has a lateral orientation to the origin of the blast source, it is possible that mechanical transmission of blast energy differs compared to other orientations. Extensive evidence has been provided that skull dynamics contribute to blast-induced TBI [62–66]. Vibration of the skull from the shock wave causes secondary brain tissue displacement and injury stems from susceptibility of the viscoelastic brain to shear forces [62, 64, 67]. Other studies speculate that vascular surge, or venous pressure pulse that is transmitted to the brain through the jugular veins after blast exposure to the thorax, is a mechanism of blast-induced brain injury [33, 68]. This mechanism could rationalize a biophysical basis of ongoing BBB disruption after BPT due to lung injury. Our BPT model also highlights the importance of cerebrovasculature due to its impedance, density gradient, and systemic connection. Multiple studies have confirmed that microcontusion and microhemorrhaging of the BBB occurs with a lower threshold of 200 kPa peak overpressure in direct cranial and lateral blast exposure models [49, 69]. Also, sudden regional-specific changes in cerebral blood flow caused by increased intracranial pressure [46, 63, 64] can cause a rapid ischemic event, contributing to hypoxic cascade later on [70]. The amygdala has been shown to be hypervascularized, indicating increased dysregulation of neural networks susceptible to vascular mechanisms [71].

Blast polytrauma is a complex injury that can encompass trauma to several specific areas of the body. For our experimental model, we focused on replicating injuries that play the largest role acutely, lung injury, and chronically, brain injury. This model is also unique due to the multiple mechanisms of injury to the brain. Mechanical insult by the blast wave on the brain has been documented by several researchers [62, 63, 66]. Though the BPT model in this study has an increased input blast overpressure compared to the mTBI model, our research group has previously shown that this relationship can be nonlinear with neuropathology [40]. To exacerbate the direct injury from blast, there is a gradual acute response of systemic hypoxia on the brain in this polytrauma model. Our group has reported that there are minimal effects on the lungs and therefore an absence of systemic hypoxia after blast-induced mTBI [35, 72]. Future studies will examine oxidative stress and susceptibility of blast-induced BBB breakdown in hypoxic environments.

### 4.5. Future Directions and Unique Therapeutic Solutions

As polytrauma incidence increases due to terrorism activities, there has been a lack of characterizing the neuropathological aspects of blast polytrauma. Understanding specific mechanisms in this unique injury mode can impact the approach to treating polytrauma. While polytraumatic injury can be complex, the time course of systemic inflammation and other systemic effects on the brain can be crucial to therapeutic intervention and prehospital management. While these findings give a general view of mechanisms present at this time point, more detail on neuroinflammation is needed to investigate specific pathways after BPT. Also, determining which components of the BBB are disrupted could point to how it is being damaged and at what point it is involved in injury pathology. MMPs have been implicated to play a major role in HIF-1*α*/VEGF cascade and BBB disruption. Examining the role of MMPs could elucidate therapeutic strategies.

In addition to the effect on acute lung trauma, the primary injury mechanisms of blast-induced TBI can be influenced by orientation of the animal within the blast tube. Acute hypoxemia can produce immediate cerebrovascular pathology. Expansion of physiology recording is needed to see how long hypoxia is present after initial injury. This could solidify hypoxia as a major concern after systemic injury. This will also give an idea of the best time window for therapeutics designed to mitigate early factors in place to aggravate injury pathology at later stages. Blood biomarkers are another way to further characterize this polytrauma, specifically targeting astrogliosis (GFAP/S100*β*), BBB breakdown (VEGF), and hypoxia (HIF-1*α*).

## 5. Conclusion

While many overlapping mechanisms in blast polytrauma coincide with that of blast-induced TBI, specific markers, such as BBB dysfunction and hypoxic factors, can play a larger role in neuropathology. For BPT, the combination of mechanically driven and hypoxic-driven neuropathology can worsen neurological outcomes. In this study, it is shown that BPT has a unique pathology and should have a different therapeutic approach compared to mTBI.
